# Prediction of single pulmonary nodule growth by CT radiomics and clinical features — a one-year follow-up study

**DOI:** 10.3389/fonc.2022.1034817

**Published:** 2022-10-28

**Authors:** Ran Yang, Dongming Hui, Xing Li, Kun Wang, Caiyong Li, Zhichao Li

**Affiliations:** ^1^ Department of Radiology, Second People’s Hospital of JiuLongPo District, Chongqing, China; ^2^ Department of Radiology, Chongqing Western Hospital, Chongqing, China

**Keywords:** pulmonary nodule, computed tomography, prediction, growth, radiomics, LASSO, logistics regression

## Abstract

**Background:**

With the development of imaging technology, an increasing number of pulmonary nodules have been found. Some pulmonary nodules may gradually grow and develop into lung cancer, while others may remain stable for many years. Accurately predicting the growth of pulmonary nodules in advance is of great clinical significance for early treatment. The purpose of this study was to establish a predictive model using radiomics and to study its value in predicting the growth of pulmonary nodules.

**Materials and methods:**

According to the inclusion and exclusion criteria, 228 pulmonary nodules in 228 subjects were included in the study. During the one-year follow-up, 69 nodules grew larger, and 159 nodules remained stable. All the nodules were randomly divided into the training group and validation group in a proportion of 7:3. For the training data set, the t test, Chi-square test and Fisher exact test were used to analyze the sex, age and nodule location of the growth group and stable group. Two radiologists independently delineated the ROIs of the nodules to extract the radiomics characteristics using Pyradiomics. After dimension reduction by the LASSO algorithm, logistic regression analysis was performed on age and ten selected radiological features, and a prediction model was established and tested in the validation group. SVM, RF, MLP and AdaBoost models were also established, and the prediction effect was evaluated by ROC analysis.

**Results:**

There was a significant difference in age between the growth group and the stable group (P < 0.05), but there was no significant difference in sex or nodule location (P > 0.05). The interclass correlation coefficients between the two observers were > 0.75. After dimension reduction by the LASSO algorithm, ten radiomic features were selected, including two shape-based features, one gray-level-cooccurence-matrix (GLCM), one first-order feature, one gray-level-run-length-matrix (GLRLM), three gray-level-dependence-matrix (GLDM) and two gray-level-size-zone-matrix (GLSZM). The logistic regression model combining age and radiomics features achieved an AUC of 0.87 and an accuracy of 0.82 in the training group and an AUC of 0.82 and an accuracy of 0.84 in the verification group for the prediction of nodule growth. For nonlinear models, in the training group, the AUCs of the SVM, RF, MLP and boost models were 0.95, 1.0, 1.0 and 1.0, respectively. In the validation group, the AUCs of the SVM, RF, MLP and boost models were 0.81, 0.77, 0.81, and 0.71, respectively.

**Conclusions:**

In this study, we established several machine learning models that can successfully predict the growth of pulmonary nodules within one year. The logistic regression model combining age and imaging parameters has the best accuracy and generalization. This model is very helpful for the early treatment of pulmonary nodules and has important clinical significance.

## Introduction

According to the glossary of terms proposed by the Fleischner Society, a pulmonary nodule is defined as an approximately rounded opacity with a diameter of less than 3 cm (1). Recently, an increasing number of pulmonary nodules have been found during screening. Studies have shown that approximately 12.0% of the US population has incidental pulmonary nodules (2). Pulmonary nodules may develop into lung cancer. A total of 2.27% of incidental pulmonary nodules developed into lung cancer during a 2-year follow-up (2). According to data from the World Health Organization (3), lung cancer was the leading cause of cancer death, with 1.8 million deaths in 2020. Early diagnosis can greatly help with treatment (4) and improve the prognosis of millions of patients.

However, for single pulmonary nodules, there are many difficulties in the selection of treatment methods and operation time. Several societies, such as The American College of Chest Physicians (5), The British Thoracic Society (6), and The Fleischner Society of the United States (7–9), have developed guidelines for the management of pulmonary nodules. The American College of Radiology has also developed a structured report template (Lung-RADS) based on the needs of diagnostic radiology practice (10). These guidelines provide recommendations for the management of pulmonary nodules according to the classification of risk factors and nodule morphology. For different types of nodules, it is recommended to carry out a second CT test at different intervals, and further treatment is determined according to the dynamic changes of nodules. The practice intervals recommended by these guidelines currently depend solely on the size of the nodules. For example, the Fleischner Society’s 2017 guideline (9) recommends review after 12 months for solid nodules smaller than 6 mm and within 3-6 months for partially solid and ground-glass nodules larger than 6 mm. If the growth of nodules can be predicted in advance, the review interval can be adjusted according to the predicted results and biopsy/surgical pathology can be conducted earlier and improve the prognosis of patients.

Conventional HRCT can reflect the size and general morphology of nodules but cannot provide depth information based on the visual information. Radiomics was proposed by Philippe Lambin in 2011. It refers to an automated and repeatable analysis that uses a high-throughput method to extract a large number of image features from radiographs (11). Since the concept of radiomics emerged, it has been widely used in the identification, grading, efficacy evaluation and prognostics of various tumors (12–15). For example, radiomics has been successfully used to distinguish benign and malignant pulmonary nodules (16, 17). Yu et al. also developed a transfer learning radiomics (TLR) model for the prediction of lymph node metastasis of papillary thyroid carcinoma and achieved high accuracy (18). However, until now, there has been no study to predict the growth of pulmonary nodules in one year using radiomics.

In this study, we intended to collect more than 200 patients with incidental pulmonary nodules and to follow up with them for one year to observe the dynamic changes in the nodules. After that, the correlation between the high-throughput features extracted by radiomics and the growth of pulmonary nodules was then analyzed. On this basis, a model was proposed to predict whether nodules are likely to grow within one year. This model can help doctors operate on dangerous nodules in time and reduce the number of re-examinations for stable nodules.

## Materials and methods

### Patients

From Jan 2020 to Dec 2021, a total of 314 patients from the Second People’s Hospital of JiuLongPo District and Chongqing Western Hospital were involved, and all of them were followed up for one year. This study was approved by the ethics committees of the two hospitals. As a retrospective analysis, the informed consent requirement was waived.

**Figure 1:**
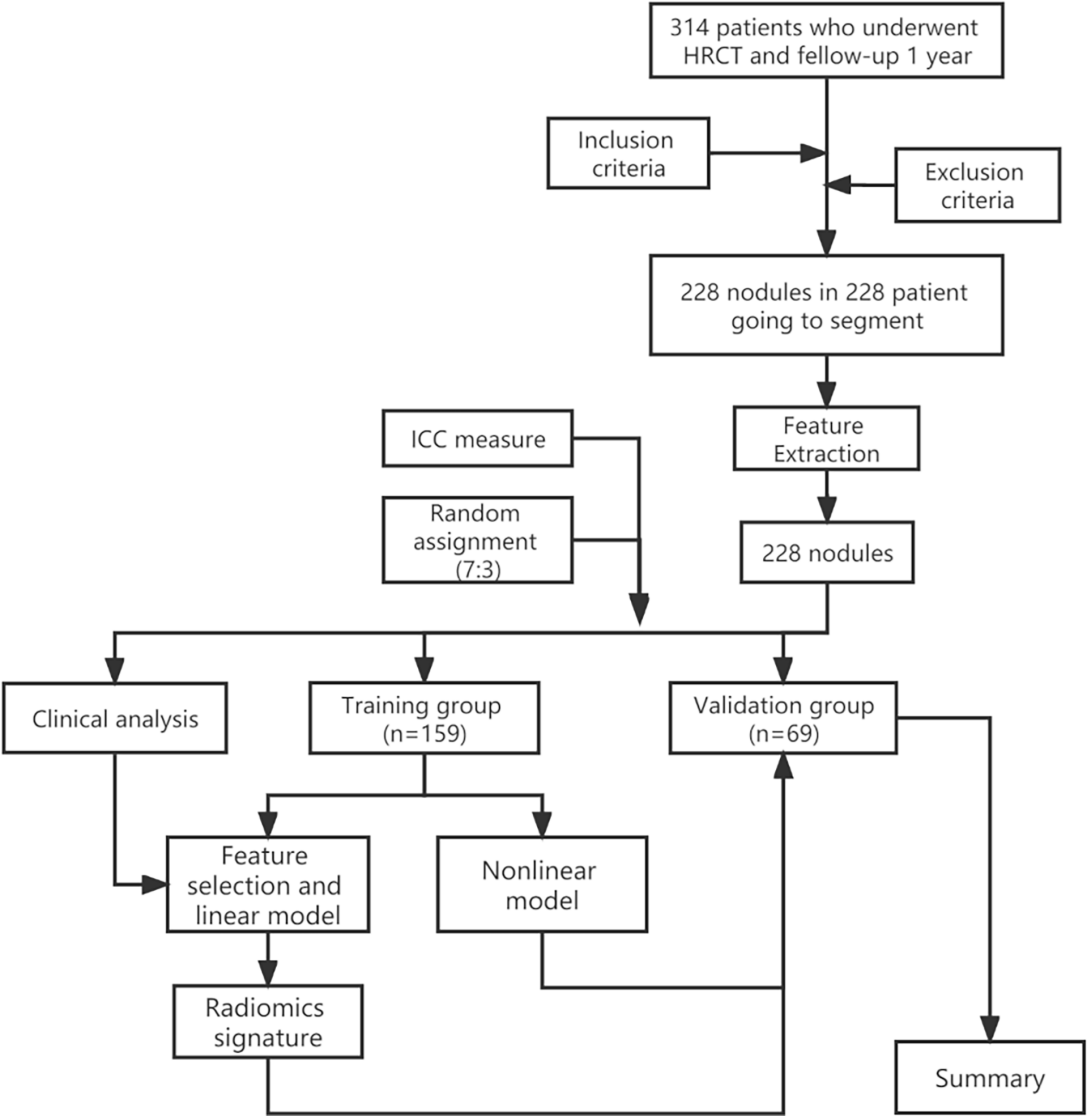
Overview workflow of this study (HRCT, high-resolution computed tomography).

The inclusion criteria were as follows: (a) patients with high-resolution chest CT images at baseline and at the one-year follow-up. (c) The nodule was solitary, and the baseline diameter of pulmonary nodules was ≥3 mm and ≤20 mm. The exclusion criteria were as follows: (a) the patient’s information was incomplete. (b) The image quality was low, (c) the nodules disappeared during follow-up, and (d) multiple pulmonary nodules were found in the baseline images. An overview of the workflow of this study is shown in **Figure 1**.

The follow-up protocol were as follows: (a) the size of the nodule was 6-8mm, and HRCT of lung scan was performed at 6-12 months. (b) The nodules were 8-20mm in size and HRCT of lung scans were performed every 3 months.

Through the exclusion criteria, 228 of 314 patients for follow-up were finally included. All patients were randomly divided into a training group and a validation group at a ratio of 7:3. The pulmonary nodules were labeled growth or stable according to whether they grew within the one-year follow-up. According to the literature (19), growing nodules were defined as nodules that increased in diameter by more than 1.8 mm in one year. Stable nodules were defined as a change in size of less than 1.8 mm over a year.

### CT scanning

The CT images were obtained on a dual source scanner (Siemens SOMATOM Drive, Siemens Healthineers, Germany), a 64-slice detector scanner (Canon Aquilion PRIME TSX-303A, Canon Medical, Japan) and a 16-slice detector scanner (Philips Brilliance 16, Philips Medical, Netherlands). The scanning parameters were as follows:

- SOMATOM Drive: tube voltage: 120 kV; tube current: automatic; detector collimation = 0.6 mm * 128; pitch: 1.2; rotation time = 0.5 s; reconstruction layer thickness: 1 mm; reconstruction matrix: 512 * 512.
- Aquilion PRIME: tube voltage: 120 kV; tube current: automatic; detector collimation = 0.5 mm * 64; pitch: 0.824; rotation time = 0.75 s; reconstruction layer thickness: 1 mm; reconstruction matrix: 512*512;
- Brilliance 16: tube voltage: 120 kV; tube current: 200-300 mAs; detector collimation = 0.75 mm * 16; pitch: 0.938; rotation time = 0.75 s; reconstruction layer thickness: 1 mm; reconstruction matrix: 512*512.

The scan area was from the thoracic entrance to the lung base, covering the whole lung. The scanning was started when the patient held their breath at the end of inhalation.

### Region-of-interest segmentation

All images were exported as Dicom files from the scanners. The DICOM images were converted to Nifft format by MRICroGL software (version: 2.1.60). The Nifft format images were imported into 3D-Slicer (an open-source software application for visualization and analysis of medical image computing data sets) (20). The regions of interest (ROIs) were independently segmented by two radiologists with more than 6 years of clinical experience. Two-dimensional ROIs were limned around the boundary of the lesions on each layer of axial CT images. Three-dimensional ROIs (volume of interest) were conducted by the accumulation of all two-dimensional region ROIs.

### Radiomics features extraction

**Figure 2:**
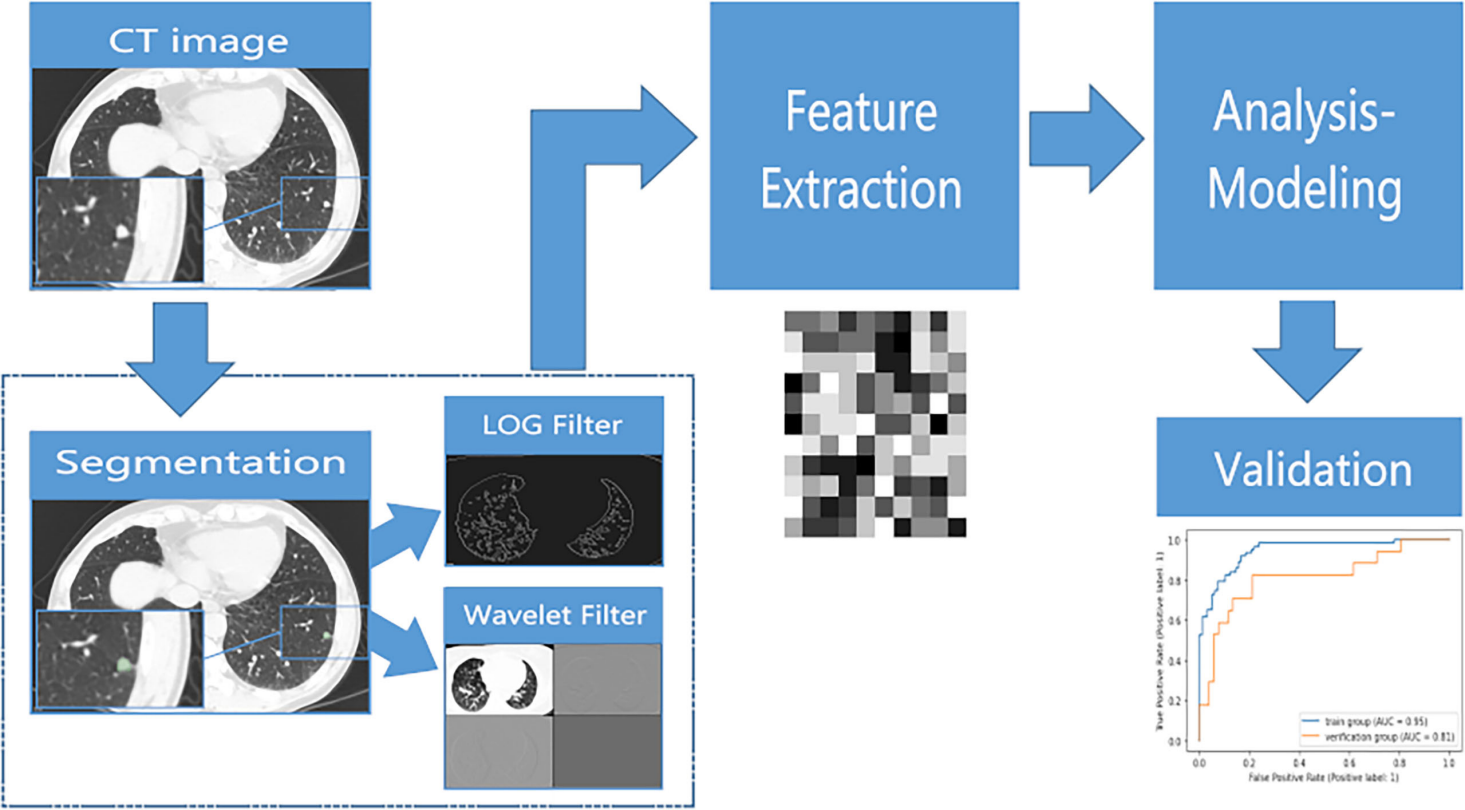
The flow chart of radiomic feature extraction and model building.

Radiomics features were extracted by an open-source python package of Pyradiomics (21). The implementation of all radiomics features followed the Imaging Biomarkers Standardization Initiative recommendations (22). This process worked on the original images, wavelet images and Laplacian of Gaussian images. A total of 1316 features were extracted. The extracted features are listed in **Supplementary Table 1**. The definitions of the texture parameters are shown on the site of Pyradimics (https://pyradiomics.readthedocs.io/en/latest/features.html). The workflow of this process is shown in **Figure 2**.

### Prediction model building

The radiomics signature was constructed in 4 steps. In step one, all radiomic feature values were normalized. In step two, the algorithm of the least absolute shrinkage and selection operator (LASSO) method was used to select the features with a nonzero coefficient. In step three, the coefficients of the features from step two were computed using multivariate logistic regression analysis. In step four, the radscore was constructed by linearly combining the coefficients of the features from the third step.

The support vector machine (SVM), random forest (RF), adaptive boosting (Adaboost), and multilayer perceptron (MLP) machine learning algorithms were used to train the model. The algorithm deployment procedure was assessed by stratified 10-fold cross-validation in the training group, which tested each model ten times to maximize the use of data and promote the accuracy of the models (23). The grid search was used to optimize the parameters of the models. The ROC areas under the receiver operating characteristic curve (AUC) and accuracy were calculated to assess the differential ability of the models. The ML algorithms were all programmed using the Python (version 3.8) machine-learning library known as scikit-learn (version 1.1) (24).

A simple threshold screening model was constructed and was compared with the method using nodule size as a basis for the follow-up in the guidelines. The length of the nodule along the X, Y and Z axes was used to calculate the average nodule length, and the average length was used as a screening index. ROC curves were calculated under SPSS using average length. The 1-specificity and sensitivity of different lengths were calculated, and then the Jorden index was calculated to find diagnostic thresholds. The average length of 6 mm from the literature (9) was also used as the threshold for predicting nodular growth. Statistical Analysis

Statistical analyses were performed using IBM SPSS Statistics 25.0. A two-sided p value < 0.05 was considered to indicate a statistically significant difference. The approximate t test was used for the intergroup comparison of continuous variables after the homogeneity test of variance. The chi-square test was used for the intergroup comparison of categorical variables. To meet the requirements of the chi-square test (R*C), the number of nodules in the left inferior lobe anterior basal segment was merged with the posterior basal segment, and the number of nodules in the right inferior lobe anterior basal segment, medial basal segment and posterior basal segment were merged. The radiomics features between the two observers were assessed for reproducibility with intraclass correlation coefficients.

## Results

### Clinical characteristics of the patients

A total of 228 nodules were finally included in the study. Eighty nodules grew in one year (growth group), and 148 nodules remained stable (stable group). The clinical characteristics of the patients in the two groups are listed in **Table 1**. The age of the stable group was 52.56 ± 12.14 and that of the growth group was 58.41 ± 14.02. An approximate t test was performed on age, as the square difference between the two groups was even (Levene’s Test F value =2.337 at p value = 0.128). There was a significant difference in age between the two groups (t value =-2.26, p value =0.025, 95% confidence interval (CI): -9.172~-2.522), and the age of the growth group was older than that of the stable group (**Supplementary Figure 1**). The sex ratios were 83:98 and 41:56 (male:female) for the stable and growth groups, respectively. There was no statistically significant difference between the two groups (χ2 = 0.329 at p value = 0.566, **Supplementary Figure 2**). The diameters of the nodules in the stable group and the growing group were 5.56 ± 1.19 mm and 7.82 ± 2.58 mm, respectively, showing a significant difference in the T test (t = -9.042 at p value < 0.001, 95% CI -2.75 ~ -1.77, **Supplementary Figures 3–5**). The chi-square test showed no significant difference in nodule location between the two groups (χ2 = 13.294 at p value = 0.425).

**Table 1:** Clinical characteristics of the patients in the training and validation cohort.

Characteristics	Growth (n = 80)	Stable (n = 148)	F value (t/χ^2^)	P value
Sexy			0.055	0.815^a^
Male	36	69		
Female	44	79		
Age	56.75 (13.832)	52.73 (12.241)	-2.26	0.025
Diameter(mm)				< 0.001
Mean ± SD	7.82 ± 2.58	5.56 ± 1.19		
Median	7.23	5.51		
Range	3.87-17.11	3.25-9.08		
Nodule Position			16.157	0.502^a^
LS1+2	11	15		
LS3	4	1		
LS4	0	5		
LS5	1	5		
LS6	7	7		
LS7+8	3	10		
LS9	5	11		
LS10	3	2		
RS1	10	27		
RS2	10	10		
RS3	5	10		
RS4	4	7		
RS5	2	4		
RS6	6	12		
RS7	0	1		
RS8	2	9		
RS9	5	9		
RS10	2	4		
Nodule Type				Not analysis
Solid	24	47		
PS	10	4		
PGG	46	97		
Morphology				Not analysis
Smooth	53	109		
Lobulated	15	29		
Spiculated	12	10		

Quantitative variables are expressed as the mean ± standard deviation. Qualitative variables are expressed as proportion. ^a^Chi-square test was used for gender and nodule position analysis. LS1+2, Left superior lobe Apical posterior segment; LS3, Left superior lobe Anterior segment; LS4, Left superior lobe Superior lingula segment; LS 5, Left superior lobe Inferior lingula segment; LS6, Left inferior lobe Superior segment; LS7+8, Left inferior lobe Anterior basal segment; LS9, Left inferior lobe Lateral basal segment; LS10, Left inferior lobe Posterior basal segment;RS1, Right superior lobe Apical segment; RS2, Right superior lobe posterior segment; RS3, Right superior lobe Anterior segment; RS4, Right middle lobe Lateral segment; RS5, Right middle lobe Medial segment; RS6, Right inferior lobe Superior segment; RS7, Right inferior lobe Medial segment; RS8, Right inferior lobe Anterior segment; RS9, Right inferior lobe Lateral segment; RS 10,Right inferior lobe Posterior segment; PS, Partly solid; PGG, Purely ground glass.

### Characteristics of the radiomics parameters

**Figure 3:**
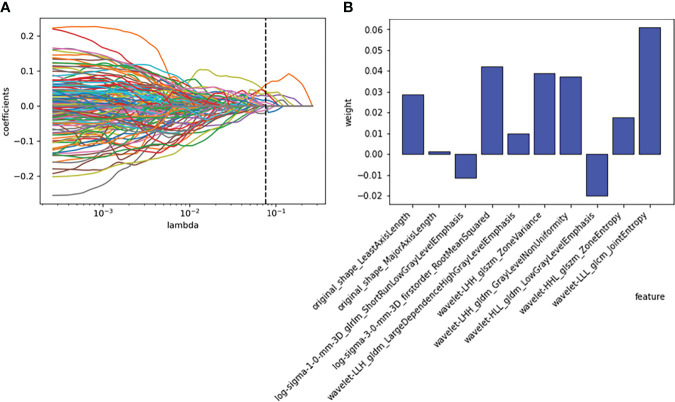
Employing the least absolute shrinkage and selection operator (LASSO) algorithm to reduce the redundancy feature. **(A)** Regression coefficient diagram of LASSO. **(B)** Features selected and their weight.

A total of 1316 features were extracted from each nodule. A total of 107 features were extracted from the original image, 465 features were extracted from the LOG filtered image, and 744 features were extracted from the wavelet filtered image. With the least absolute shrinkage and selection operator (LASSO), ten features were selected to form a radiomics signature for predicting the growth of nodules. The ten selected features with their contribution coefficients are shown in **Figure 3**. They included two shape-based features, one gray-level- cooccurrence-matrix (GLCM), one first-order feature, one gray-level-run-length-matrix (GLRLM), three gray-level-dependence-matrix (GLDM) and two gray-level-size-zone-matrix (GLSZM).

### Linear prediction model

**Figure 4:**
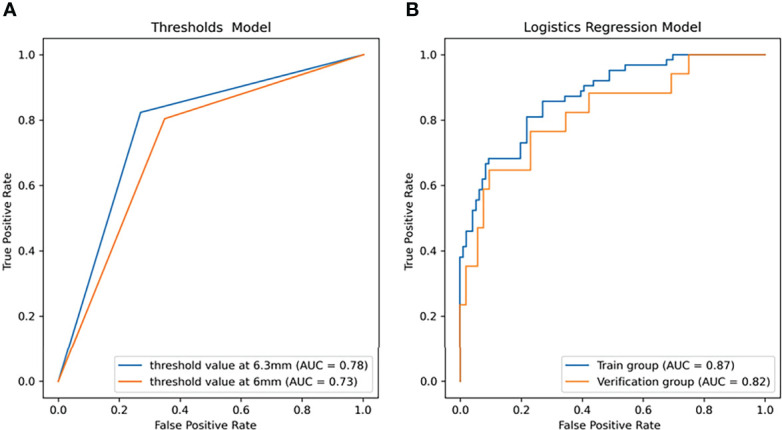
The receiver operator characteristic (ROC) curves of the linear models for predicting the growth of the nodules within one year. **(A)** ROC curve of the threshold prediction model (area under the ROC curve (AUC) = 0.73 as threshold at 6 mm, AUC = 0.77 as threshold at 6.3 mm). **(B)** ROC curve of logistic regression (LR) (AUC = 0.87 in the training group, AUC =0.82 in the validation group).

**Figure 5:**
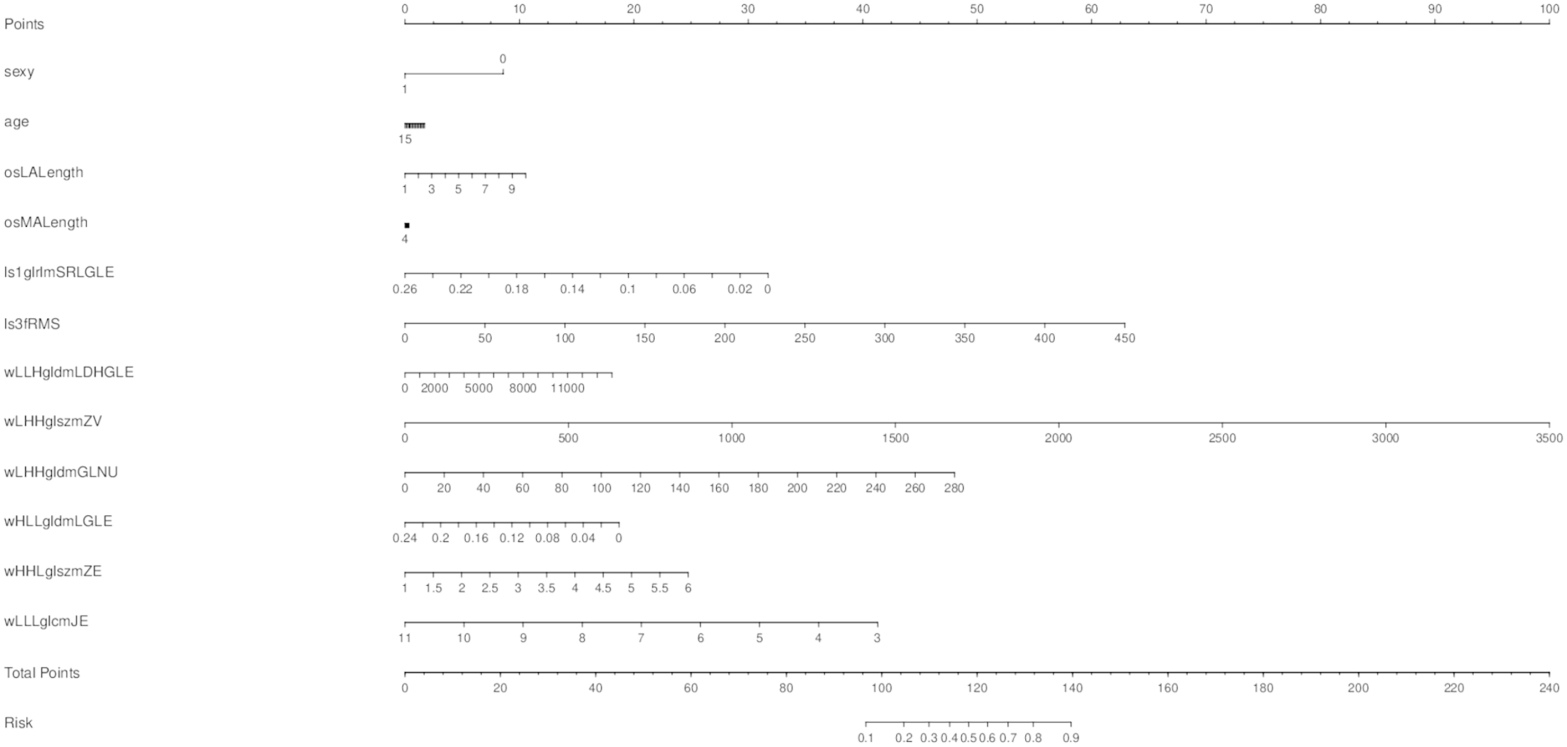
A nomogram was made to predict the one-year growth of single pulmonary nodules.

The ten radiomics features selected by LASSO and the clinical signature (age) were combined to establish a classification model by logistic regression. The AUC and accuracy attained by the combined model on the training group and validation group were 0.87 (95% CI: 0.74–0.98), 0.82, 0.82 (95% CI: 0.68–0.95) and 0.84, respectively (**Figure 4B**). The relationship between the predicted value and the true value is shown in the line chart in the **Supplementary Materials** (**Supplementary Figure 6**). The established logistics classification formulation is stated in the **Supplementary Material**, and the nomogram is described in **Figure 5**.

While using the nodule diameter line length as the screening threshold, in the training group, the diagnostic threshold for mean length was 6.3 mm (sensitivity: 0.778, specificity: 0.771, AUC: 0.81). With 6.3 mm as the threshold, the accuracy and AUC in the validation group were 0.754 and 0.777, respectively, but when 6 mm was used as the threshold to predict growth in all 278 patients, the accuracy and AUC were 0.705 and 0.728, respectively (**Figure 4A**).

### Nonlinear prediction models

In this study, four nonlinear methods were trained to predict the growth of the nodules, including support vector machine (SVM), random forest (RF), adaptive boosting (Adaboost), and multilayer perceptron (MLP). The ROC curves of the four nonlinear models in the training group and validation group are shown in **Figure 4**, and the classification reports of these models are listed in **Table 2**.

**Table 2:** The classification report of the different models on the validation group.

Model	Precision	Recall	F1-score	Accuracy
Logistic Regression				0.84
Stable	0.89	0.90	0.90	
Growth	0.69	0.65	0.67	
SVM				0.81
Stable	0.88	0.87	0.87	
Growth	0.61	0.65	0.63	
MLP				0.68
Stable	0.88	0.67	0.76	
Growth	0.41	0.71	0.52	
RF				0.74
Stable	0.84	0.81	0.82	
Growth	0.47	0.53	0.50	
Adaboost				0.78
Stable	0.88	0.83	0.85	
Growth	0.55	0.65	0.59	

The precision, recall, F1-score of the logistic regression, SVM, MLP and Adaboost model in the validation group. SVM, Support vector machine; RF, Random Forest; MLP, Multilayer perceptron.

**Figure 6:**
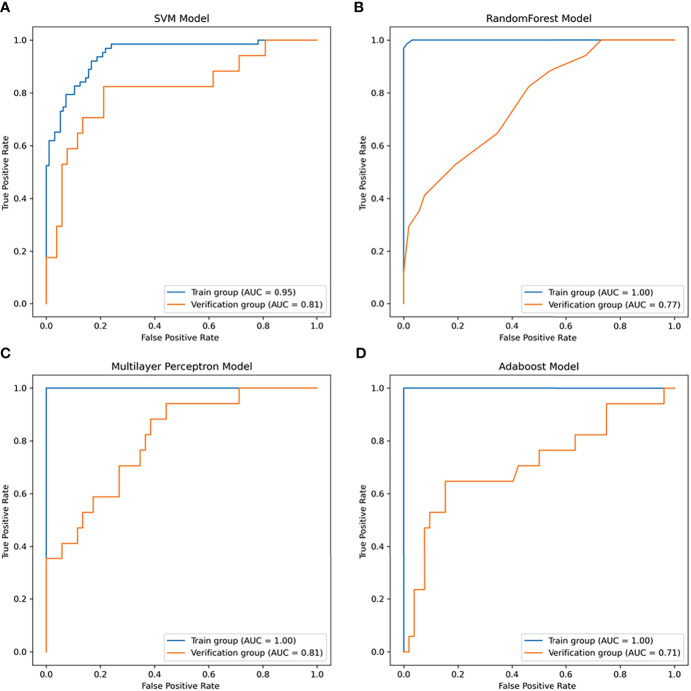
The receiver operator characteristic (ROC) curves of the nonlinear models for predicting the growth of the nodules within one year. **(A)** ROC curve of the SVM model (area under the ROC curve (AUC) = 0.95 in the training group, AUC =0.81 in the validation group). **(B)** ROC curve of the random forest (RF) model (AUC = 1.0 in the training group, AUC =0.77 in the validation group). **(C)** ROC curve of the multilayer perceptron (MLP) model (AUC = 1.0 in the training group, AUC =0.81 in the validation group). **(D)** ROC curve of the Adaboost model (AUC = 1.0 in the training group, AUC =0.71 in the validation group).

In the training group, the AUC of the SVM model was 0.95 (95% CI: 0.82-0.99, **Figure 6A**), the accuracy rate was 0.86, the AUC of the RF model was 1.00 (95% CI: 0.76-1.0, **Figure 6B**), the accuracy rate was 0.99, the AUC of the MLP model was 1.00 (95% CI: 1.00: 0.79-1.0, **Figure 6C**), and the accuracy was 1.00. The AUC of the Adaboost model was 1.00 (95% CI: 0.84-1.0, **Figure 6D**), and the accuracy was 1.00. In the validation group, the AUC of the SVM model was 0.81 (95% CI: 0.64-0.89, **Figure 6A**), the accuracy rate was 0.81, the AUC of the RF model was 0.77 (95% CI: 0.660-0.83, **Figure 6B**), the accuracy rate was 0.74, and the AUC of the MLP model was 0.81 (95% CI: 0.69-0.92, **Figure 6C**). The AUC of the Adaboost model was 0.71 (95% CI: 0.62-0.76, **Figure 6D**), and the accuracy was 0.78.

## Discussion

Pulmonary nodules are very common, and it is difficult to accurately predict their growth. Tumor growth kinetics (TGK) have usually been used for the prediction of tumor growth in the past. It is generally considered to have three well-defined phases: the first (lagged phase) is associated with tumor establishment in the host; the second stage (log or exponential) is associated with rapid tumor growth; and the third stage (stationary phase) shows slow growth of the tumor and gradual convergence to the final volume (25). To describe tumor growth, the exponential growth model (26), linear growth model and Gompertzian growth model (27) have been proposed. These models require pathological data of tumors, such as cell lines, cell surface diffusion, and cell proliferation, which cannot be obtained before surgery.

In clinical practice, CT follow-up is of great clinical significance to help manage pulmonary nodules without pathological information. The Fleischner Society of the United States, the American College of Chest Physicians, the British Thoracic Society, and the American College of Radiology have published their guidelines for the management of nodules based on CT findings to help physicians develop an effective follow-up protocol. However, even among the most widely applied Fleischner guidelines, there was considerable heterogeneity in the choice of nodule treatment in clinical practice (8). Additionally, the CT findings adopted by these guidelines were gross morphology, which was limited in information. Previous studies have shown that radiomics features can be used to analyze the biological and pathophysiological information of lung cancer and provide rapid and accurate noninvasive biomarkers for its diagnosis, prognosis and treatment response monitoring (28). This study was the first to use radiomics tools to predict single pulmonary nodule growth within one year. The results showed that our model performs well in both the training group and the validation group. This model could help to develop a follow-up plan for uncertain pulmonary nodules and reduce the over treatment of nodules in clinical practice.

In this study, five different machine learning methods were used to develop prediction models of whether pulmonary nodules would increase within one year. In general, the growth of nodules was related to gender, adhesion, location, size, and characteristics of nodules (29). The size and characteristics (such as solid, subsolid, ground glass, and spiculated) in the guidelines were gross changes, and high-throughput radiomics features could decompose these features into more detailed texture features to determine more nuanced information. These features included size and shape-based features, first-order features of the image gray histogram, second-order features of image voxel relations, such as gray-level cooccurrence matrix (GLCM), run length matrix (RLM), size zone matrix (SZM) and neighborhood gray tone difference matrix (NGTDM), texture features extracted by wavelet and Gaussian Laplacian filter, etc. (22). These high-dimensional data contained information reflecting the underlying pathophysiology (30), which can be revealed by quantitative image analysis (31, 32). In this study, the 1316 radiomics features extracted from the CT images were reduced to ten features with the LASSO algorithm. The ten features and their weights are shown in **Figure 3B**. Among them, the morphological features LeastAxisLength and MajorAxisLength reflected the nodule size, which corresponded to the nodule diameter adopted in the guidelines (5–7, 9). In a previous study of portal phase expansive versus infiltrative tumor growth front, wavelet_LHH_glrlm_ShortRun-LowGrayLevelEmphasis was considered to be the best predictor of tumor growth (33, 34). The pathological association of textural features derived from gray-level cooccurrence matrices (GLCMs) has been proven and applied to the diagnosis of breast cancer (35). The GLSZM and GLDM features could reflect tumor heterogeneity and homogeneity (36).

Generally, age, sex and nodule location are related to whether a nodule is benign or malignant (7, 9, 37), but whether these factors could predict the growth of a nodule within one year is unclear. In this study, the average age of the patients with enlarged nodules was older than that of the patients with stable nodules at the 1-year follow-up, and the difference was statistically significant. These results indicated that age was an independent predictor of nodule growth (38). There was no significant difference in sex or nodule location between the two groups. This finding was inconsistent with literature reports that women and nodules in the upper lobe of the right lung were risk factors for lung cancer (39). A possible reason was that this study focused on nodular growth rather than benign or malignant nodules, and the growth curves of benign and malignant nodules partially overlapped (40).

In this study, the logistic regression model has the best AUC and accuracy compared to the SVM, RF, MLP and AdaBoost models. It can help doctors predict whether the nodules will grow after one year and has important clinical significance. In previous studies, logistic regression models have been used to predict the malignant degree of solitary pulmonary nodules (41), showing good predictive performance. The nonlinear ML algorithm can deal with multidimensional features and identify some underlying patterns from data that are not linear or polynomial. Previously, Jiang Yuming et al. found that an SVM model can predict the survival rate of gastric cancer patients (42). Mitra Montazeri found that the random forest model is a useful tool for survival prediction and medical decision-making of breast cancer (43). QZ et al. successfully used the AdaBoost model to predict local prostate cancer recurrence (44). MLP models have also been used to predict mortality in elderly patients with hip fractures (45). In this study, the LR model obtained the best AUC and F1 scores in the validation group among the five models, so it was selected to construct the prediction formula and nomogram. The SVM, RF, MLP and AdaBoost models had high AUC and accuracy in the training group but showed low performance in the validation group. Therefore, overfitting may exist and could affect the generalization of the model. According to previous studies, the more complex the model is, the overfit is more likely, the more parameters need to be adjusted, and more samples are needed to learn (46). Therefore, in this study, these models performed worse than the LR models.

In conclusion, in this study, we found that the logistical regression model combining high-resolution CT-derived radiomics and age could accurately predict whether a lung nodule will increase after one year. It has great potential clinical value in helping clinicians develop diagnostic and treatment strategies.

The study has several limitations. First, the sample size was relatively small due to the strict inclusion/exclusion criteria, nearly one-third of the patients were lost to follow-up, and there may have been a potential selection bias. Second, patients with multiple nodules were not included in the analysis. Third, in the model construction, only the imaging features of high-resolution CT plain scans were used, and other imaging data were not considered. In the future, more patients need to be followed up to verify the validity of the model, and different imaging technologies, such as CT enhancement and MRI, should be combined to further improve the prediction efficiency of the model.

## Data availability statement

The original contributions presented in the study are included in the article/**Supplementary Material**. Further inquiries can be directed to the corresponding authors.

## Ethics statement

The studies involving human participants were reviewed and approved by the ethic committee of the second people’s hospital of JiuLongPo district and the Chongqing western hospital. Written informed consent to participate in this study was provided by the participants’ legal guardian/next of kin.

## Author contributions

RY, DH, XL and KW collected the relevant data, and RY and ZL analyzed the data. DH, and ZL wrote this manuscript. ZL put forward the study topic and revised the manuscript. All authors read and approved this manuscript.

## Funding

This study was supported by grants from the Science and Health Joint Medicine research project of Chongqing (including traditional Chinese medicine) (grant number 2022MSXM140).

## Acknowledgments

The authors thank Siemens Healthcare, Philips Healthcare, and Canon Medical for their kind support.

## Conflict of interest

The authors declare that the research was conducted in the absence of any commercial or financial relationships that could be construed as a potential conflict of interest.

## Publisher’s note

All claims expressed in this article are solely those of the authors and do not necessarily represent those of their affiliated organizations, or those of the publisher, the editors and the reviewers. Any product that may be evaluated in this article, or claim that may be made by its manufacturer, is not guaranteed or endorsed by the publisher.
