# Supplementary material for: Prediction of single pulmonary nodule growth by CT radiomics and clinical features — a one-year follow-up study
Source: Front Oncol. 2022 Oct 28;12:1034817. doi: 10.3389/fonc.2022.1034817 (PMC9650464; doi:10.3389/fonc.2022.1034817)
Supplement: Supplementary file 1 [file DataSheet_1.docx]

Supplementary Material

The formula for calculating the probability of nodular growth:

p(y=1|xi)= 1/(1-exp(-1* (-1.85266563 + age * 0.002617 + original_shape_LeastAxisLength * 0.015074 - original_shape_MajorAxisLength * 0.073091 - log-sigma-1-0-mm-3D_glrlm_ShortRunLowGrayLevelEmpasis * 10.656316 + log-sigma-3-0-mm-3D_firstorder_RootMeanSquared * 0.013779 + wavelet-LLH_gldm_LargeDependenceHighGrayLevelEmphasis * 0.000149 + wavelet-LHH_glszm_ZoneVariance * 0.002701 + wavelet-LHH_gldm_GrayLevelNonUniformity * 0.022875 - wavelet-HLL_gldm_LowGrayLevelEmphasis * 3.362045 + wavelet-HHL_glszm_ZoneEntropy * 0.601211 - wavelet-LLL_glcm_JointEntropy * 0.412040 ))).


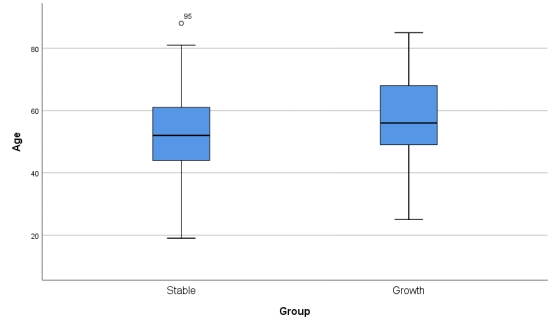


**Supplementary Figure 1.** Age composition of the growing and stable groups.


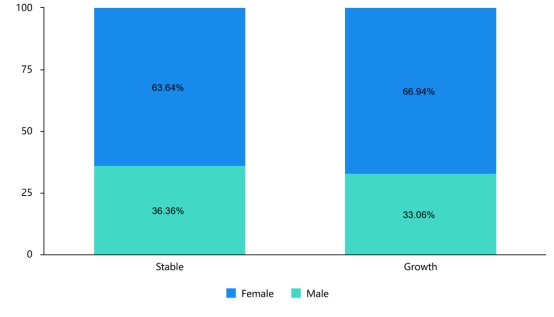


**Supplementary Figure 2.** Stacked Bar Chart of gender composition of growth group and control group.


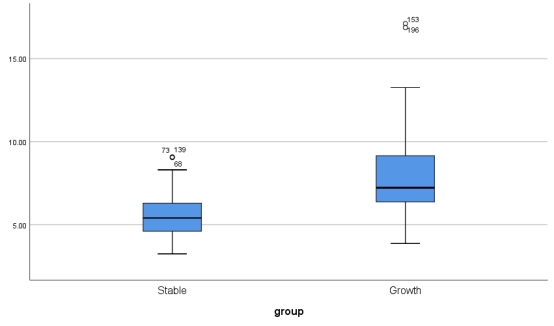


**Supplementary Figure 3.** Box plots of the diameter of nodules in the stable group and growth group.


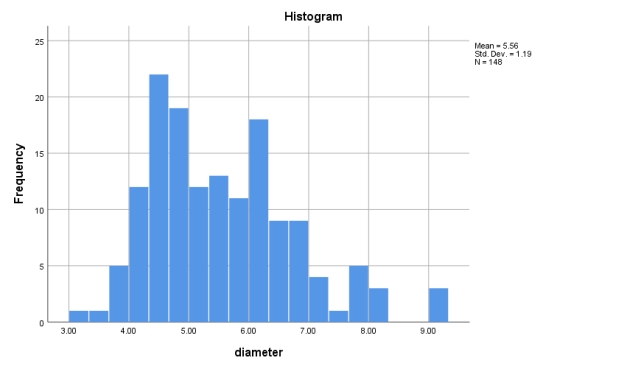


**Supplementary Figure 4.** Diameter distribution of nodules in stable group.


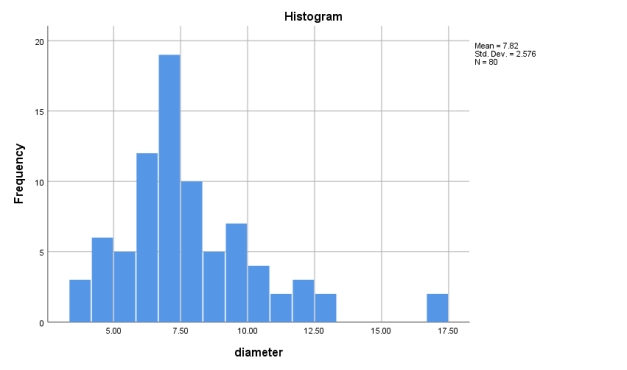


**Supplementary Figure 5.** Diameter distribution of nodules in growth group.


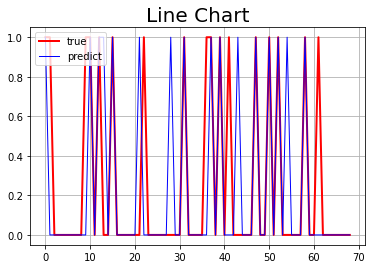


**Supplementary Figure 6.** The line chart of the relationship between the predicted value and the true value in the validation group.


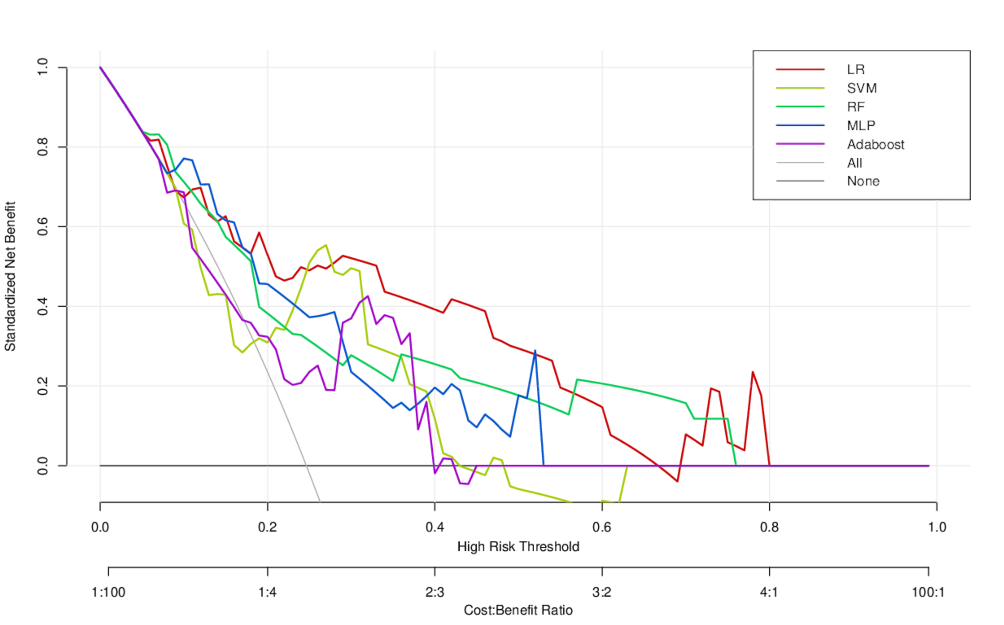


**Supplementary Figure 7.** Decision Curve Analysis of the five models.

| **Supplementary TABLE 1 \|** Features extracted from CT Image | |
| --- | --- |
| Features Class | Feature Amount |
| Original Image | 107 |
| Shape-based | 14 |
| First Order Statistics | 18 |
| Gray Level Cooccurence Matrix | 24 |
| Gray Level Run Length Matrix | 16 |
| Gray Level Size Zone Matrix | 16 |
| Neighbouring Gray Tone Difference Matrix | 5 |
| Gray Level Dependence Matrix | 14 |
| LoG filtered Image | 465 |
| First Order Statistics | 90 |
| Gray Level Cooccurence Matrix | 120 |
| Gray Level Run Length Matrix | 80 |
| Gray Level Size Zone Matrix | 80 |
| Neighbouring Gray Tone Difference Matrix | 25 |
| Gray Level Dependence Matrix | 70 |
| Wavelet filtered Image | 744 |
| First Order Statistics | 144 |
| Gray Level Cooccurence Matrix | 192 |
| Gray Level Run Length Matrix | 128 |
| Gray Level Size Zone Matrix | 128 |
| Neighbouring Gray Tone Difference Matrix | 40 |
| Gray Level Dependence Matrix | 112 |

| **Supplementary TABLE 2 \|** The diameter of nodules in the lost follow-up group and the enrolled group | | | | |
| --- | --- | --- | --- | --- |
| Group | Number | Mean | Std. Deviation | Std. Error Mean |
| Enrolled | 21 | 5.8781 | 2.21978 | .48440 |
| Lost | 21 | 6.3216 | .96497 | .21057 |

| **Supplementary TABLE 3 \|** Independent samples test of nodules in the lost follow-up group and the enrolled group | | | | | | | | | | |
| --- | --- | --- | --- | --- | --- | --- | --- | --- | --- | --- |
|  | | Levene's Test for Equality of Variances | | t-test for Equality of Means | | | | | | |
|  |  | F | Sig. | t | df | Sig. | Mean Diff. | Std. Error Diff. | 95% CI of the Difference | |
|  |  |  |  |  |  |  |  |  | Lower | Upper |
| Diameter | Equal variances assumed | 11.875 | .001 | -.840 | 40 | .406 | -.44353 | .52819 | -1.51104 | .62397 |
|  | Equal variances not assumed |  |  | -.840 | 27.298 | .408 | -.44353 | .52819 | -1.52673 | .63966 |
